# A decade of *Alzheimer’s Research & Therapy*: reflections on the past, present, and future

**DOI:** 10.1186/s13195-020-00629-y

**Published:** 2020-05-30

**Authors:** Douglas Galasko, Philip Scheltens

**Affiliations:** 1 University of California, San Diego, USA; 2 Alzheimer Center, Amsterdam UMC, The Netherlands

## A brief history of the journal’s development and increasing impact

*Alzheimer’s Research & Therapy* was born after a series of conversations at an ICAD meeting (now known as AAIC) and some arm twisting between representatives of BioMed Central and the first Editors, Doug Galasko and Gordon Wilcock. We were persuaded about the novelty and value of an entirely online journal with a translational focus in Alzheimer’s disease and managed to enlist an outstanding Editorial Board to provide guidance. From the outset, our goal was to promote research from basic, translational, and clinical researchers, and to have an international focus, with broad representation of research interests among our advisors. We tried to maintain high standards for publication and as rapid a turnaround of reviews as we could achieve.

We quickly realized the need for an Editor with basic research expertise and were delighted when Todd Golde agreed to join in 2010. Todd’s deep knowledge base, energy, and inspiration contributed greatly to the quality, breadth, and rigor of basic science that we have published. Unfortunately, Todd has decided to step down as Editor as of 2020, and we would like to call out the outstanding contributions he has made to shaping the journal during a decade of service. In 2013, Gordon Wilcock retired as Editor, and we were fortunate to persuade Philip Scheltens to accept the role. Philip’s commitment and high standards have helped to enhance the performance of *Alzheimer’s Research & Therapy* and to shape its direction.

Our impact factor has steadily increased to 6.142, as of the 2018 Journal Citation Report, as shown in the figure. We hope to see this trend continue with the release of the 2019 report in June. The journal has been consistently ranked in the first quartile for Clinical Neurology as well as Neuroscience journals. As can be seen in an excerpt from the most recent Publisher’s Report in Table 1, we have published papers from authors from across the globe. North America, Europe/UK, Australia, China, Taiwan, and South Korea predominate, but Africa, India, South America, and Middle Eastern submissions and publications are represented. Because one of our areas of focus is on new therapeutic development for Alzheimer’s, we have had, and welcome, submissions from Industry. Although some publications are outliers, we have maintained an average time from submission to first decision of 60 days and are constantly reaching out to broaden our pool of reviewers.

**Table 1:** Top ten most accessed articles over the last 12 months

Article name	Corr. author	DOI	Accesses
Cerebral microbleeds: overview and implications in cognitive impairment	Sergi Martinez-Ramirez et al.	10.1186/ALZRT263	17,167
Testing a MultiTEP-based combination vaccine to reduce A-beta and tau pathology in Tau22/5xFAD bigenic mice	Hayk Davtyan et al.	10.1186/S13195-019-0556-2	14,241
Alzheimer’s disease drug-development pipeline: few candidates, frequent failures	Jeffrey L Cummings et al.	10.1186/ALZRT269	7209
Herbal therapy: a new pathway for the treatment of Alzheimer’s disease	Jinzhou Tian et al.	10.1186/ALZRT54	6715
Alzheimer’s disease prevention: from risk factors to early intervention	Marta Crous-Bou et al.	10.1186/S13195-017-0297-Z	6230
Souvenaid in the management of mild cognitive impairment: an expert consensus opinion	Jeffrey Cummings et al.	10.1186/S13195-019-0528-6	5890
Comprehensive treatment of dementia with Lewy bodies	Brendon P Boot	10.1186/S13195-015-0128-Z	5734
The clinical characteristics of dementia with Lewy bodies and a consideration of prodromal diagnosis	Paul C Donaghy et al.	10.1186/ALZRT274	5031
Effects of spermidine supplementation on cognition and biomarkers in older adults with subjective cognitive decline (SmartAge)—study protocol for a randomized controlled trial	Miranka Wirth et al.	10.1186/S13195-019-0484-1	4854
Alcohol use and dementia: a systematic scoping review	Jürgen Rehm et al.	10.1186/S13195-018-0453-0	4714

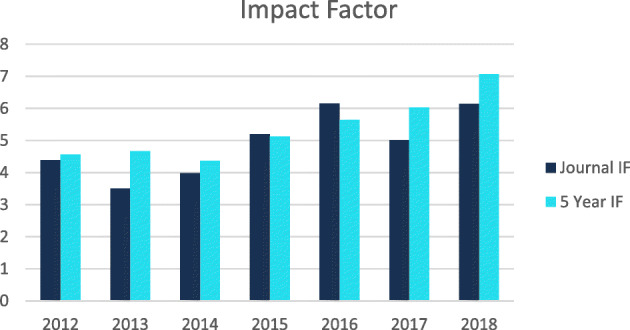



(2018 Journal Citation Reports® (Clarivate Analytics, 2018))

## A selection of papers that reflect the success of the journal

The table lists the top 10 accessed articles in the past year. This reflects precisely the scope of our journal: covering the breadth of the field from a translational point of view with a clear focus on therapy. Also, the highly accessed reviews on dementia with Lewy bodies and chronic traumatic encephalopathy demonstrate how research on other disorders that may cause dementia complements the landscape of Alzheimer’s research.

## Future directions

Following this brief summary of our 10 years of existence, it is time to reflect: what is good, what can be done better, and what should we reconsider? And how should the past decade of research into Alzheimer’s influence these ideas? This decade has been marked by the frustration of therapeutic failures in Alzheimer’s that have led some to question the amyloid hypothesis as a central dogma, the emergence of tau-related therapeutic efforts, exciting developments in biomarkers—including brain imaging and CSF- and blood-based markers—and increasing awareness of the importance of neuroinflammation. A biomarker-based classification of Alzheimer’s that enables detection and can support intervention studies before the development of cognitive decline is receiving extensive investigation. Digital biomarkers including passive sensors, wearables, and device-based testing are increasingly being studied and deployed. Neuropathological and biomarker studies are demonstrating the concept that multiple pathologies contribute to neurodegeneration and cognitive decline. Extrinsic factors that may contribute to brain development, resilience, reserve, and vulnerability are being widely studied and have been conceptualized as the exposome. Efforts to understand Alzheimer’s and related disorders from a genetic, molecular, and -omic standpoint are burgeoning. These have led to renewed attention to preclinical models of disease, from cell-based models (including human models derived from iPS-cell technology) to animal models. New methods of protein analysis, including cryo-electron microscopy, are providing insights into the structure of proteins, including aggregated proteins emblematic of Alzheimer’s and related disorders. Drug discovery and translation now include small molecules, immunological therapies, and biological approaches, as well as efforts to investigate the impacts and biology of lifestyle interventions.

Against this background, we believe that the overall scope of the journal should be retained: translational research into Alzheimer’s, with the additional emphasis that we should also include and learn from other types of dementia, neurodegenerative, or otherwise. Translational research encompasses clinical perspectives, biomarkers, genetics, epidemiology, pathology, mechanisms of disease relevant to translation, and therapy across all stages of development, and in particular research with interactions or translation between these areas! We are interested in cognition and cognitive reserve and on early changes along the Alzheimer continuum, as evidenced by a recent thematic series. We are less interested in observational research (for example, epidemiology in healthy aging subjects), basic research on aging, animal models outside of translational studies, or cognitive/behavioral studies in healthy (elderly) individuals. We are interested in meta-analyses and studies of large healthcare databases only if they have substantial novelty and impact.

Original data papers, reviews, and thematic series remain the mainstay of our publications. Topics for thematic series have been and will be developed with extensive input from the Editorial Board. Some recent examples are shown in the table (see Table 2). We have published relatively few debates, opinions, or commentaries and do not intend to change that policy, although we may make an occasional exception, when a paper evokes a serious debate or has novel/controversial findings.

**Table 2:** Thematic series over the years

**2019**	
Subject cognitive decline	
**2018**	
Resilience in dementia	
**2015**	
Innate immunity in Alzheimer’s disease	
The impact of acute and chronic medical disorders on accelerated cognitive decline	
**2014**	
Cerebral multi-morbidity of the aging brain	
Lewy body dementia	
**2013**	
Immunotherapy in Alzheimer’s disease	
Tau-based therapeutic strategies	
Abeta catabolism	
Traumatic brain injury	
Cognitive enhancers for ageing and Alzheimer’s disease	
Peripheral biomarkers	
**2012**	
The new FTD mutation on chromosome 9	
**2011**	
Early-onset dementia	
Amyloid Imaging	
**2010**	
Failed clinical trials	
Prevention trials	

Next to us as Editors-in-Chief, we have invited Associate Editors (see Table 3), including one Biostatistical Advisory Editor, to help us assess the growing number of original manuscripts that we receive. The Associate Editors will help us to cover the broad range of expertise needed to handle diverse research topics. They will help in assessing papers, searching for reviewers, and handling papers up until the final decision which is made by the Editors-in-Chief. By asking them to help us, we aim to be able to handle more submissions with greater timeliness. This expanded Editorial Team will exercise the option to reject a paper outright if it is judged outside of the scope of the journal, is of too low quality to send out for review, and overlaps with or repeats another published paper. Except for the statement that the manuscript falls below the standards of Alz R&T, the editors will not provide a specific reason for this immediate rejection. By doing this, we hope this will help authors to seek a more appropriate home for their submission without unnecessary delays. We may add further Associate Editors in the future.

**Table 3:** The Editorial Team, April 2020

**Editors-in-Chief**	
Philip Scheltens, *Amsterdam UMC, Netherlands*	
Doug Galasko, *University of California, San Diego, USA*	
**Associate Editors**	
Michael Heneka, *University Hospital Bonn, Germany*	
Bruno Imbimbo, *Chiesi Farmaceutici, Italy*	
Seth Love, *University of Bristol, UK*	
Jose Luis Molinuevo, *BBRC, Spain*	
Rik Ossenkoppele, *Amsterdam UMC, Netherlands*	
Charlotte Teunissen, *Amsterdam UMC, Netherlands*	
Pieter Jelle Visser, *Amsterdam UMC, Netherlands*	
**Statistical Advisory Editor**	
Suzanne Hendrix, *Pentara Corporation, USA*	

We remain committed to as rapid a turnaround time as possible from submission, through reviews and to first Editorial decision. To accomplish this, we need many reviewers with diverse knowledge and skills, and we deeply appreciate the helpful responses we have had from many colleagues to serve in this capacity over the years. As with everyone in the field, we are all very busy, but if authors want their papers to be reviewed by capable reviewers, we ask our authors in return also to agree to review. We are all in this together!

Biomed Central is now part of the Springer Nature family. This merger has allowed the journal to consistently stay ahead in serving our community of authors and readers, not least through initiatives such as a venture into offering an integrated pre-print server through a recent partnership with *In Review*. We continue to explore ways to foster and improve communication and interactions through an online journal, including the use of social media such as the @AlzheimersRes and @BioMedCentral twitter feeds to distribute links to manuscripts and Calls for Papers, and interviewing authors to describe more about the work.

Finally, since 2013, we have been affiliated with Alzheimer’s Disease International (ADI) and through our partnership have opened up *Alzheimer’s Research & Therapy* publications to a new audience. We have promoted ADI resources and activities on the webpage and have published reports or reviews of ADI events in the journal. In turn, ADI have championed the journal at their conferences and shared highlights from our publications with their associated researcher groups.

We are delighted to announce that we will soon partner with Alzheimer’s Research UK! The Editors are highly enthusiastic about this because it will open up a new audience for our journal, may attract new authors and reviewers, will allow us to explore scientific publications on events organized by ARUK, and will provide a solid backbone by an organization dedicated to Alzheimer’s disease research.

We thank our authors and reviewers for their continuing support for our journal to the benefit of the next decade of better and more groundbreaking research to finally defeat the disease which at the core of our minds.

